# Mental Health and Substance Use among Bisexual Youth and Non-Youth in Ontario, Canada

**DOI:** 10.1371/journal.pone.0101604

**Published:** 2014-08-11

**Authors:** Lori E. Ross, Greta R. Bauer, Melissa A. MacLeod, Margaret Robinson, Jenna MacKay, Cheryl Dobinson

**Affiliations:** 1 Health Systems & Health Equity Research Group, Centre for Addiction and Mental Health, Toronto, Ontario, Canada; 2 Epidemiology & Biostatistics, Schulich School of Medicine and Dentistry, The University of Western Ontario, London, Ontario, Canada; The University of New South Wales, Australia

## Abstract

Research has shown that bisexuals have poorer health outcomes than heterosexuals, gays, or lesbians, particularly with regard to mental health and substance use. However, research on bisexuals is often hampered by issues in defining bisexuality, small sample sizes, and by the failure to address age differences between bisexuals and other groups or age gradients in mental health. The Risk & Resilience Survey of Bisexual Mental Health collected data on 405 bisexuals from Ontario, Canada, using respondent-driven sampling, a network-based sampling method for hidden populations. The weighted prevalence of severe depression (PHQ-9≥20) was 4.7%, possible anxiety disorder (OASIS≥8) was 30.9%, possible post-traumatic stress disorder (PCL-C≥50) was 10.8%, and past year suicide attempt was 1.9%. With respect to substance use, the weighted prevalence of problem drinking (AUDIT≥5) was 31.2%, and the weighted prevalence of illicit polydrug use was 30.5%. Daily smoking was low in this sample, with a weighted prevalence of 7.9%. Youth (aged 16–24) reported significantly higher weighted mean scores on depression and post-traumatic stress disorder, and higher rates of past year suicidal ideation (29.7% vs. 15.2%) compared with those aged 25 and older. The burden of mental health and substance use among bisexuals in Ontario is high relative to population-based studies of other sexual orientation groups. Bisexual youth appear to be at risk for poor mental health. Additional research is needed to understand if and how minority stress explains this burden.

## Introduction

Population-based studies in several countries indicate health disparities associated with sexual orientation, wherein sexual minorities (lesbians, gays or bisexuals) have poorer health outcomes than heterosexuals, especially on measures of mental health and substance use [1]–[3]. In a recent analysis of Canadian population-based data, 17.1% of self-identified sexual minority individuals reported a current mood disorder, compared to only 6.9% of heterosexual respondents [3]. Significant disparities have also been reported in rates of cigarette smoking [4]–[5], suicide attempts, anxiety disorders, and substance dependence [6]. The mental health burden of anticipated and experienced discrimination associated with minority sexual orientations (i.e., minority stress) has been proposed to explain these health disparities [7].

Although most of the early population-based investigations on sexual minority mental health collapsed bisexuals together with lesbians and/or gays, recent studies that examined bisexuals independently indicate that bisexual people tend to have the poorest mental health outcomes of all sexual orientation groups. In studies that compare bisexuals to their gay or lesbian peers, bisexuals report higher rates of anxiety [8]–[9], depression [8]–[9], and self-harm behaviour [9]–[10]. Studies comparing bisexual women with lesbians likewise found elevated rates of anxiety [11], depression [11]–[12], poor self-rated mental health [13], and suicidality [12]–[13] for bisexual women. Suicidality data from the Canadian Community Health Survey (CCHS Cycle 2.1) are particularly striking: bisexual women reported nearly a six-fold increase in odds of lifetime suicidality relative to heterosexuals, while lesbians had nearly a four-fold increase in odds [14]. Among men, the increased risk for suicidality compared to heterosexuals was nearly seven-fold for bisexuals and four-fold for gay men [15].

Significant gaps remain in our knowledge about bisexual mental health. As reviewed by Kaestle & Holz Ivory, health research on bisexuality is limited by the fact that few studies examine bisexuals independent of other sexual minority groups, and there is an over-reliance on convenience samples with poor representation of females and youth [16]. Mental health outcomes in population-based data tend to be limited in their scope and focused on single-item measures of mood and anxiety disorder [8], [14]. Moreover, some research has used recent (e.g. past-year) behavioural measures of bisexuality, which have been demonstrated to serve as poor proxies for self-identified bisexuality or for lifetime bisexual behaviour, conflating the effects of sexual orientation with those of sex partner number: one must have two partners to be labelled as behaviourally bisexual, yet only one to be labelled as gay/lesbian, or heterosexual [17]. A more comprehensive picture of bisexual health, including a variety of mental health and substance use outcomes for a population reflecting the use of this term in communities, is necessary in order to understand if and how the minority stress framework applies to bisexuals.

Because the number of bisexuals in most population-based studies has been relatively small, few studies report how health outcomes among bisexuals might vary across age. Lesbian, gay and bisexual youth and youth who report same-sex romantic attraction have higher rates of depressive symptoms and suicidality than their heterosexual peers [18]–[19]. However, as noted by the Institute of Medicine in their recent report on the health of lesbian, gay, bisexual and transgender people, very little research has examined health and health care for bisexual youth specifically [20].

In this study we aim to address these knowledge gaps by producing population estimates based on a large sample of bisexual individuals aged 16 and older in Ontario, Canada's most populous province. We focus our analysis on seven mental health and substance use outcomes, disaggregating our findings according to age.

## Methods

### Study Sample

Data were collected as part of the Risk & Resilience Survey of Bisexual Mental Health. Sampling was undertaken using respondent-driven sampling (RDS) with an internet-based English-language survey. RDS is a method of chain-referral sampling in which participants are able to recruit an additional number of eligible new participants (here up to 10), and recruitment proceeds through social networks. In this way, researchers are able to make inferences about a population that cannot be sampled using traditional population-based methods such as random sampling [21]–[22]. To date, empirical validation studies indicate that point estimates calculated using RDS are generally similar to population proportions [23]–[24].

Recruitment networks were tracked using a numerical coupon system, so that structural characteristics of the network could be used in statistical analysis to account for the non-randomness in social networks. Eligible participants were those who identified themselves as attracted to individuals of more than one sex/gender, were 16 years of age or older, and residents of the province of Ontario, Canada (n = 405). Based on previous community-based research by our team [25], we opted for this inclusive attraction-based definition of bisexuality, which community members identified as most accurately reflecting use of the term within bisexual communities. The entire sample identified with this broad definition. In addition, most (61.6%) of the sample personally identified as ‘bisexual’, often in combination with other identity terms (e.g., queer, pansexual, fluid).

### Measures

Participants self-completed the survey, which included a wide range of items related to demographics, mental health and substance use.

**Sexual orientation** was queried using a single check all that apply item with 13 fixed options plus a write-in option (“You don't have an option that applies to me. I identify as (specify)”). The 13 fixed options were developed in consultation with our community advisory committee to reflect sexual orientation identities currently in use among bisexual communities in Ontario, and included: Ambisexual, Asexual, Biaffectionate, Bisensual, Bisexual, Fluid, Heteroflexible, Homoflexible, Omnisexual, Pansexual, Queer, Questioning, and Not Sure.

**Sex at birth** was assessed with a single item, “What was the sex you were assigned at birth?”, with two options, “Male” and “Female”.

**Gender identity** was queried using a single check all that apply item with 8 fixed options plus a write-in option (“You don't have an option that applies to me. I identify as (specify)”). The 8 fixed options were developed in consultation with our community advisory committee to reflect gender identities current in Ontario, and included: 2-Spirited, Bigendered, Crossdresser, Genderqueer, Man, Trans Man, Trans Woman, And Woman.

**Racial, ethnic, and cultural identity** was assessed using a single check all that apply item including 13 categories adapted from the question included in Statistics Canada's long form census [27], plus a write-in option (“Other: please specify”).

**Age** was assessed by subtracting birth year from the year in which the survey was completed. We labelled those aged 16–24 as “youth” and those aged 25 years or older as non-youth.

Depression was measured using the **Patient Health Questionnaire Depression Scale (PHQ-9)** [27]. Depressive symptoms are indicated by nine items on a 4-point Likert scale, which measures the frequency of symptoms ranging from 0 = *not at all* to 3 = *nearly every day*. Scoring indicates depression severity. This measure has been used in research with gay and trans samples [28]–[29], and has demonstrated validity and test-retest reliability [27]. We examined both mean PHQ-9 scores and categories of depression severity using cut-off scores suggested by Kroenke et al. [27]. In this study, the PHQ-9 had high internal consistency with a Cronbach's alpha of 0.87.

Anxiety was measured using the **Overall Anxiety Severity and Impairment Scale (OASIS)** [30]. This scale has 5 items and measures anxiety symptoms on a 5-point Likert scale ranging from 0 = *no anxiety* to 4 = *constant*. We are unaware of OASIS being utilized with LGBT samples, but the scale has strong evidence of validity and reliability [30]–[31]. We examined both mean OASIS scores and probable anxiety disorder as indicated by OASIS scores≥8 [30]. In our study, the OASIS had high internal consistency with a Cronbach's alpha of 0.87.

Symptoms of post-traumatic stress disorder (PTSD) were measured using the **PTSD Checklist – Civilian Version (PCL-C)** [32]. The scale includes 17 items that are measured on a 5-point Likert scale ranging from 1 = *not at all* to 5 = *extremely*. The PCL-C has strong support for validity and reliability [33] and has been successfully used with LGBT participants. In our study, the PCL-C had very high internal consistency with a Cronbach's alpha of 0.92. We examined both mean PCL-C scores and probable PTSD using two previously published cut-off scores: ≥44 and ≥50 [33].

The **Alcohol Use Disorders Identification Test (AUDIT)** was developed by the World Health Organization to screen for excessive drinking [34]. The test includes 10 questions that are measured on a 5-point Likert scale with varying anchors of frequency (e.g., 0 = *never*, 4 = *daily or almost daily*). A cut-off of 4 or more is often recommended for women, and a cut-off of 5 or more is usually recommended for men. Considering the inclusion of trans-identified people in our study, a cut-off of 5 was selected for both men and women to indicate problem drinking. The AUDIT has extensive evidence of validity and reliability across gender, age and culture [34]. Chronbach alpha's are generally reported in the 0.80's [35]. In our study, the Cronbach's alpha demonstrated good internal consistency with a value of 0.74.

**The Drug Use Disorders Identification Test- Extended Version (DUDIT-E)** was developed to assess drug use problems among persons attending treatment programs. The DUDIT-E includes 11 items that assess drug use patterns and possible drug-related problems. Researchers are currently exploring the psychometrics of the DUDIT-E and there is support for the strength of this measure [36]–[37]. In our study, internal consistency was high with a Cronbach's alpha of 0.82. Given that the DUDIT-E was developed for use in populations with high levels of substance use, responses were categorized for this analysis into no past year drug use, single drug use, and polydrug use.

**Suicidality** was measured by using: “Have you ever seriously considered committing suicide or taking your own life?”; “Has this happened in the past 12 months?”; “Have you ever attempted to commit suicide or tried taking your own life?”; and “Did this happen in the past 12 months?” These questions were developed and validated by Statistics Canada for the Canadian Community Health Survey Cycle 4.1 [26]. Skip patterns were forward-filled to provide past-year measures for the entire study sample.

**Tobacco Use**. Tobacco smoking was measured by self-report using the following item: “At the **present time**, how often do you smoke cigarettes?” This item was also developed and validated by Statistics Canada for the Canadian Community Health Survey Cycle 4.1 [26].

### Ethics Statement

All participants indicated their consent to participate in the survey by selecting a button reading “I have read and understood the information on the web page, and agree to participate in this research survey” prior to providing any survey data. Participants aged 16 and 17 years of age consented on their own behalf; consent of their next of kin, caretakers or guardians was not sought due to the potentially sensitive knowledge that would be disclosed through the consent process (i.e., bisexual identity). The study was reviewed and approved by the Research Ethics Board of the Centre for Addiction & Mental Health (CAMH). Following the example of the Hospital for Sick Children, the CAMH Research Ethics Board considers that those who are 16 years of age and up may provide their own consent to participate and that children under age 16 may assent but need the formal consent of their parents or guardians.

### Statistical Analysis

Data were cleaned and coded in SAS version 9.3 [38]. Respondent-Driven Sampling Analysis Tool (RDSAT) version 6.0 [39] was used to estimate weighted frequencies for the networked Ontario bisexual population [21]–[22]. Associated 95% confidence intervals were produced using a modified form of bootstrapping [40], wherein resampling is conducted via recruitment chains with 10,000 resamples using an enhanced data-smoothing algorithm. Since chi-square tests cannot be conducted using this method, variance recovery methods were used to produce confidence intervals around the differences in proportions (not shown) [41], and p-values were generated from these [42].

To produce weighted means and associated 95% confidence intervals for continuous measures among Ontario bisexuals, individualized weights were generated using RDSAT for several variables (PHQ-9, OASIS, PCL-C, and AUDIT). These were merged into the SAS data file and used with adjustment for clustering by shared recruiter to produce weighted means, 95% confidence intervals, and t-tests for differences between means.

## Results

**Figure 1:**
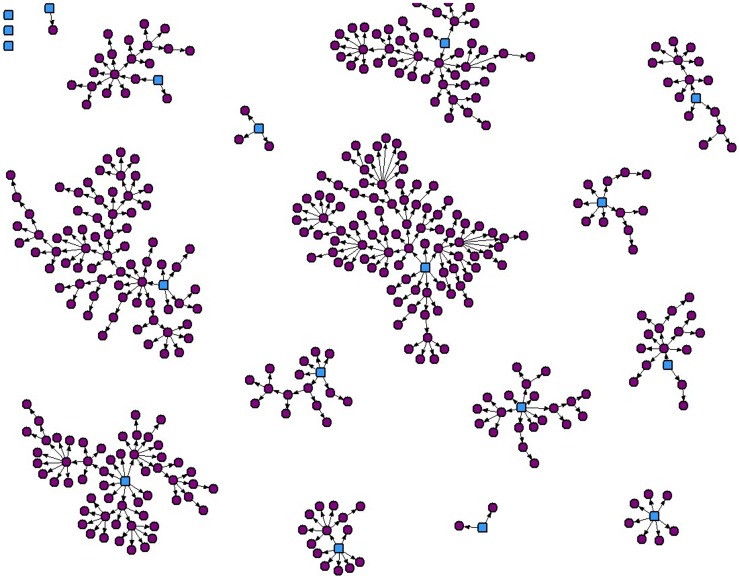
Recruitment network diagram for respondent-driven sampling survey of 405 bisexuals in Ontario, Canada. Blue = seed. Purple = recruit.

Of 18 seeds, 15 generated at least one additional participant, with a resulting final sample of 405. A maximum of nine waves of recruitment was achieved beyond the original seeds. The recruitment structure is displayed in Figure 1. Total tree sizes ranged from 2 to 93. A total of 23% of the sample was generated from the largest tree, and two-thirds of the sample (66.2%) was generated from a combination of the four largest trees.

Weighted estimates of demographics for networked bisexual individuals in Ontario are presented in Table 1. Our estimates suggest that the networked population of bisexual people was young, with an estimated 75% being under 35 years of age. Networked bisexuals were also estimated to be predominantly female assigned at birth (69.7% vs. 30.3% male assigned at birth). A substantial proportion identified as trans or a related term; for example, an estimated 6.6% identified their gender as ‘genderqueer’, 1.7% as ‘trans man’ and 0.5% as ‘trans woman’. Finally, the networked population of bisexual people in Ontario was estimated to be predominantly white (85.2% indicated a white identity or background, either alone or in combination with other backgrounds) and born in Canada (84.8%), though substantial proportions indicated racialized identities or backgrounds (e.g., Aboriginal: 6.3%; Black: 7.6%).

**Table 1:** Estimated demographic characteristics of bisexual Ontarians (N = 405).

	Youth			Non-Youth			Total		
		Weighted frequencies			Weighted frequencies			Weighted frequencies	
Characteristic	N	%	95% CI	N	%	95% CI	N	%	95% CI
Age									
16 to 24 years	99	33.6	(23.0, 44.8)	0	–	–	99	33.6	(23.0, 44.8)
25 to 34 years	0	–	–	177	41.3	(32.6, 51.2)	177	41.3	(32.6, 51.2)
35 to 44 years	0	–	–	79	15.1	(8.5, 22.2)	79	15.1	(8.5, 22.2)
45 to 54 years	0	–	–	34	7.2	(2.6, 12.0)	34	7.2	(2.6, 12.0)
55+ years	0	–	–	12	2.8	(0.2, 8.3)	12	2.8	(0.2, 8.3)
Sex at Birth									
Female	77	81.1	(71.2, 90.4)	222	66.0	(53.4, 75.0)	302	69.7	(60.2, 77.5)
Male	22	18.9	(9.6, 28.8)	80	34.0	(25.0, 46.6)	103	30.3	(22.6, 39.8)
Gender Identity*									
Woman	63	62.7	(43.7, 74.5)	195	64.2	(54.2, 74.7)	261	64.2	(55.9, 73.0)
Man	22	20.0	(10.0, 31.7)	79	32.0	(23.1, 43.5)	101	27.7	(20.4, 35.9)
Genderqueer	14	10.8	(2.5, 15.8)	39	5.3	(3.1, 7.9)	53	6.6	(3.5, 9.7)
2-spirited	6	4.8	(1.4, 9.0)	17	2.8	(0.7, 4.7)	23	3.2	(1.2, 5.0)
Trans man	6	2.4	(0.3, 4.1)	13	1.3	(0.4, 3.0)	19	1.7	(0.7, 2.9)
Trans woman	2	0.4	(0.0, 1.2)	3	0.7	(0.0, 2.2)	5	0.5	(0.1, 1.2)
Identity not named above‡	13	14.0	(5.4, 23.7)	25	5.3	(2.6, 8.2)	43	8.5	(4.6, 13.0)
Racial, Ethnic or Cultural Identity*									
White	92	88.3	(75.3, 95.4)	254	83.4	(74.5, 90.0)	349	85.2	(78.0, 90.5)
Aboriginal/First Nations	7	7.4	(0.9, 17.3)	30	5.2	(2.0, 8.4)	38	6.3	(3.2, 10.6)
Black	5	6.8	(1.1, 17.9)	15	6.0	(0.6, 12.7)	21	7.6	(2.6, 13.5)
South Asian	1	0.4	(0.0, 1.6)	9	2.2	(0.3, 4.4)	10	1.7	(0.4, 3.4)
Latin American	2	5.2	(0.0, 16.3)	7	3.2	(0.7, 7.8)	9	3.4	(0.8, 7.5)
Chinese	4	2.6	(0.1, 7.4)	3	0.9	(0.0, 2.4)	7	1.5	(0.4, 3.0)
Identity not named above†	9	12.3	(4.4, 22.3)	21	6.6	(2.5, 12.6)	30	7.6	(3.7, 12.7)
Born in Canada	89	92.3	(85.0, 97.3)	249	81.8	(73.6, 88.9)	341	84.8	(78.3, 90.2)
Education (highest level)									
High school or less	22	44.3	(18.0, 48.7)	15	5.5	(1.3, 12.8)	38	17.9	(8.4, 22.9)
Some or completed college/university	69	55.7	(51.4, 82.0)	170	75.4	(64.1, 83.1)	242	70.2	(63.4, 80.1)
Completed graduate or professional degree	0	–	–	80	19.1	(12.4, 28.6)	80	11.9	(8.0, 18.6)
Household income									
Less than $10,000	22	24.1	(13.6, 35.0)	17	4.6	(1.7, 7.7)	39	9.0	(5.4, 14.4)
$10,000 to $19,999	21	19.2	(11.6, 31.2)	45	14.4	(8.4, 21.1)	68	16.0	(11.2, 21.7)
$20,000 to $29,999	7	4.8	(0.9, 9.5)	49	15.6	(9.5, 21.6)	56	12.1	(7.8, 17.1)
$30,000 to $39,999	12	12.4	(4.0, 22.9)	29	11.6	(5.2, 19.7)	41	11.7	(6.5, 17.5)
$40,000 to $59,999	9	15.1	(3.9, 27.5)	58	20.0	(14.5, 31.9)	67	18.1	(11.8, 25.3)
$60,000 to $79,999	7	5.6	(1.5, 9.3)	43	17.0	(10.2, 26.3)	52	14.7	(9.3, 21.3)
$80,000 to $100,000	7	11.8	(3.2, 22.9)	21	7.5	(2.2, 12.3)	28	9.3	(4.5, 14.5)
Greater than $100,000	9	7.0	(2.8, 13.2)	34	9.2	(3.0, 14.4)	43	9.0	(4.4, 13.7)
Relationship Status*									
Married or partnered	20	20.1	(10.6, 31.8)	144	47.6	(36.1, 56.7)	165	39.0	(30.5, 46.8)
Single	38	47.9	(34.6, 58.8)	70	28.7	(21.7, 39.9)	110	35.6	(28.9, 44.0)
Multiple partners	18	6.9	(2.8, 11.2)	132	28.2	(20.5, 35.7)	151	22.2	(16.9, 27.7)
Region of Ontario									
Eastern Ontario	30	34.9	(17.1, 50.0)	37	21.7	(11.1, 34.3)	67	21.8	(11.6, 33.5)
Central Ontario	11	14.6	(2.8, 29.4)	39	12.9	(5.9, 20.3)	52	15.3	(8.2, 21.4)
Metropolitan Toronto	41	37.3	(21.6, 56.6)	169	49.8	(38.2, 64.9)	212	45.8	(34.0, 56.9)
Southwestern Ontario	12	9.9	(1.7, 22.5)	39	11.7	(3.3, 20.1)	51	12.5	(5.7, 22.5)
Northern Ontario	4	3.2	(0.5, 7.9)	12	3.8	(0.6, 8.4)	16	4.6	(1.9, 9.1)

*Percentages do not total 100% as participants could select more than one option.

‡ In addition to the gender identities given in the table, a total of 17 people identified as ‘bigendered’, 9 people as ‘crossdresser’, and 17 people provided a gender identity in the write-in field that could not appropriately be re-categorized into one of these options (e.g., agendered).

† The table provides frequencies for racial, ethnic and/or cultural groups with which 7 or more total participants identified.

Smaller number of participants identified as Arab, Filipino, Japanese, Korean, Southeast Asian, and West Asian, and a total of 18 participants provided a write-in response that could not be appropriately re-categorized into one of these options (e.g., Jewish).

Weighted prevalence estimates for the seven mental health and substance use outcomes are presented in Table 2. The weighted mean prevalence of severe depression as indicated by PHQ-9 scores≥20 was 4.7% for the total sample. Possible anxiety disorder, as indicated by OASIS scores≥8, was more common, with a weighted prevalence of 30.9%. Using the more conservative cut-off of PCL-C scores≥50, the weighted prevalence of PTSD in this sample was 10.8%, and the weighted prevalence of past year suicide attempt was 1.9%.

**Table 2:** Weighted mental health and substance use outcome estimates for bisexual Ontarian youth and non-youth (N = 405).

	Age			
Measure	16–24 years	≥25 years		Total
	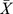 or % (95% CI)	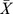 or % (95% CI)	p-values	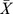 or % (95% CI)
**Depression**				
PHQ-9 ( 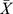 )	9.176 (8.018, 10.334)	6.787 (5.728, 7.846)	0.0038	7.495 (6.544, 8.446)
Severity (%)				
Minimal (1–4)	13.3 (10.9, 25.3)	30.9 (21.4, 41.5)	0.0129	23.8 (16.4, 29.3)
Mild (5–9)	45.6 (29.2, 53.1)	38.3 (26.9, 49.2)	0.3802	40.7 (32.5, 49.7)
Moderate (10–14)	28.1 (15.6, 36.4)	17.9 (11.2, 27.2)	0.1309	22.9 (17.9, 31)
Mod. Severe (15–19)	11.3 (7.3, 22.0)	6.3 (2.2, 13.3)	0.3102	7.8 (4.5, 12.5)
Severe (20–27)	1.7 (0.0, 3.5)	6.5 (0.5, 13.9)	0.1756	4.7 (1.0, 8.9)
**Anxiety**				
OASIS ( 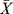 )	6.133 (5.179, 7.087)	5.466 (4.704, 6.228)	0.2721	5.808 (5.125, 6.491)
Anxiety disorder (%≥8)	38.5 (26.2, 50.0)	26.5 (17.4, 35.1)	0.1132	30.9 (23.7, 37.7)
**Suicidality**				
Suicidal ideation, past yr (%)	29.7 (18.3, 38.7)	15.2 (9.2, 21.3)	0.0180	18.8 (13.7, 24.4)
Suicide attempt, past yr (%)	5.1 (0.7, 8.4)	1.8 (0.0, 3.0)	0.1242	1.9 (0.6, 3.7)
**Posttraumatic stress disorder**				
PCL-C ( 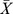 )	35.626 (32.246, 39.007)	31.135 (28.984, 33.285)	0.0275	32.495 (30.585, 34.406)
PTSD present (%≥44)	26.1 (14.1, 37.4)	15.5 (9.7, 23.2)	0.1241	17.7 (12.3, 23.4)
PTSD present (%≥50)	11.6 (5.3, 19.5)	10.8 (4.9, 17.7)	0.8701	10.8 (6.2, 15.2)
**Substance use**				
AUDIT ( 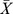 )	3.919 (3.345, 4.493)	3.848 (3.416, 4.280)	0.7795	3.862 (3.492, 4.232)
Problem drinking (%≥5)	32.6 (20.3, 42.4)	30.4 (21.5, 40.6)	0.7657	31.2 (24.8, 39.7)
Daily smoker (%)	8.2 (2.1, 15.7)	7.7 (4.1, 11.4)	0.8981	7.9 (4.9, 11.5)
Drug use (%)				
No past-year use	57.2 (42.7, 69.4)	55.6 (45.6, 65.7)	0.8510	55.5 (47.9, 64.0)
Single drug use	18.6 (9.1, 30.0)	11.8 (5.7, 20.6)	0.3015	14.0 (8.7, 20.3)
Polydrug use	24.1 (14.7, 36.6)	32.7 (23.1, 40.8)	0.2300	30.5 (23.2, 37.2)

PHQ-9: The Patient Health Questionnaire's Depression Scale.

OASIS: Overall Anxiety Severity and Impairment Scale.

PCL-C: PTSD Checklist – Civilian Version.

AUDIT: Alcohol Use Disorders Identification Test.

CI: Confidence Interval.

With respect to alcohol consumption, weighted mean AUDIT scores for the total sample were 3.86, below our cut-off of 5 for problematic consumption. However, using this cut-off, the weighted prevalence of problem drinking was 31.2% for the total sample. Rates of daily smoking were very low, with a weighted prevalence of 7.9%. Finally, with respect to use of other drugs, the weighted prevalence for single drug use (very predominantly cannabis) was 14%, and polydrug use (predominantly cannabis in combination with a variety of other substances including amphetamines, barbiturates, club drugs, cocaine, hallucinogens, inhaled drugs, and opiates) was 30.5%.

When estimates for the networked population of bisexual youth vs. non-youth were considered, youth had poorer outcomes for most mental health and substance use indicators (see Table 2). The difference between weighted estimates for youth and non-youth was statistically significant for weighted mean PHQ-9 scores (9.2 vs. 6.8 points, p<0.005), the weighted proportion with “minimal” depression (13.3% vs. 30.9%, p<0.05), the weighted proportion of past year suicidal ideation (29.7% vs. 15.2%, p<0.05), and weighted mean PCL-C scores (35.6 vs. 31.1 points, p<0.05). No significant differences were found between youth and non-youth for any of our indicators of anxiety or regarding substance use.

## Discussion

To our knowledge, this study represents the first RDS sample specifically of bisexual people undertaken. While we used the term “bisexual” for convenience, the inclusion criterion of attraction to more than one sex/gender allowed for a broad sample of individuals who did not identify solely as monosexual (e.g. gay, lesbian, heterosexual). While RDS provides opportunities to adjust for known biases in social network structure and has been shown to reach individuals who would be inaccessible through venue or other convenience sampling methods, some limitations of network-based sampling need to be acknowledged. Completely non-networked individuals (those not connected to a single other bisexual, or those who are not ‘out’ as bisexual to others in their networks) will not be included, and it remains possible that biases not tied directly to personal network size may have impacted the sample [24]. We thus cannot say with certainty that our results are valid, and there is a total absence of true population data for comparison.

Prevalence estimates for most mental health and substance use outcomes investigated in this study were higher than those reported in the general population. For example, a Canadian study of over 3000 individuals recruited through random digit dialing reported a depression prevalence of 8.4% for PHQ-9 scores≥10[43]; in this study, the weighted prevalence of PHQ-9 scores≥10 (i.e., moderate depression or greater) was 35.4%. This is consistent with studies that have used other indicators of depression: for example, using population-based data from the Canadian Community Health Survey, 11.4% of bisexual men reported a mood disorder that had been diagnosed by a health professional, compared to 4.0% of heterosexual men. For women, the rates were 25.2% for bisexuals compared to 7.5% for heterosexuals [8].

Similarly, our rates for past year suicidal ideation and attempt are substantially higher than those previously reported in population studies. In our data, the weighted prevalence of past year suicidal ideation among non-youth was 15.2%, and 1.8% for past year suicide attempt. Among adults (≥18 years and older) in the United States, 3.7% reported suicidal ideation in the past year, and 0.5% reported a suicide attempt [44]. Similarly, 29.7% of youth in our sample reported past year suicidal ideation, with 5.1% reporting a past year suicide attempt. Using data from the US National Comorbidity Survey- Adolescent Supplement, Husky et al. found that 3.6% of youth aged 13–18 reported past year suicidal ideation with no plan or attempt, and 1.9% reported a past year suicide attempt [45]. Again, these disparities are consistent with other population-based studies: CCHS data from 2003 reported a lifetime suicidality rate of 34.8% for bisexual men compared to 7.4% for heterosexual men [15]. For women, the corresponding figures were 45.4% for bisexual women and 9.6% for heterosexual women [14]. Finding that suicidality was higher in our study than others may reflect a time trend. In a province-wide school-based study of adolescents in British Columbia, Canada, suicidal ideation and attempts remained stable for heterosexual adolescents, and decreased dramatically for gay males, but increased substantially between 1992 and 2003 for lesbians and for bisexual male and female teens [18]. It is unclear whether a similar time trend exists for bisexuals beyond adolescence, or whether it has continued beyond 2003. While post-traumatic stress disorder is less often assessed in population-based surveys, on the basis of US population-based epidemiological surveys, a lifetime prevalence of 10.1% has been reported for the general population [46]. This is consistent with the weighted prevalence of 10.8% derived using the more conservative PCL-C cut-off of 50.

With respect to smoking, it is notable that rates of daily smoking were very low (7.9% of the total weighted sample), relative to either the general population or other studies of sexual minority individuals. In the general Canadian population, 16% of individuals aged 15 and older are reported to be daily smokers; this is lower than the average rate among OECD countries [47]. Daily smoking rates among sexual minority individuals are generally higher than among heterosexuals. For example, in a population-based study of adults in California, 22.2% of lesbians and 22.6% of bisexual women reported daily smoking compared to rates of 9.1% in the general population of women; 19.0% of gay men and 16.2% of bisexual men reported daily smoking compared to 13.9% of men in the general population [48].

With respect to alcohol and illicit drug use, however, we report higher rates than figures previously reported for the Canadian population. For example, in the 2011 Canadian Community Health Survey 19.0% of the population aged 12 and older reported consuming 5 or more drinks per occasion at least monthly [49], compared to our weighted estimate of 31.2% for problem drinking. With respect to illicit drug use, 2002 Canadian Community Health Survey data indicated that 87.4% of Canadians reported no past year illicit drug use [50], relative to only 55.5% among bisexual people in this study. Rates of both single and polydrug use were also higher in this study than in the general Canadian population (14.0% vs. 10.2% and 30.5 vs. 2.4% for single and polydrug use, respectively) [50].

Our finding that bisexual identity is associated with elevated rates of not only one, but several different poor health outcomes is consistent with the hypothesis that experiences of stigma, prejudice and discrimination (i.e., minority stress) may be important contributors to these disparities [7]. As Schwartz and Meyer point out, “social stress theory speaks to the causal effect of social statuses on the totality of mental health outcomes, not on specific disorders” [51]. Although little research has evaluated the discrimination experiences of bisexual people, the studies that do exist indicate that bisexual people often experience multiple forms of minority stress [25], [52]. In other words, bisexual people, like gay and lesbian people, may experience homophobia associated with their same-sex relationships and attractions; however in addition, they may also experience biphobia associated with their bisexual identity and/or relationship history. Further, discrimination may come not only from heterosexual individuals and institutions, but also from gay or lesbian individuals and institutions [53]–[54]. In this context, our team has previously proposed that bisexual people, as a group, may experience more minority stress than lesbian/gay people, and that this may explain the pronounced mental health disparities associated with bisexual identities specifically [25]. Additional research, involving both between- and within-group comparisons, is needed to test this hypothesis.

Relative to other sexual minority identities, bisexuality tends to be endorsed among younger relative to older age groups, particularly among women [8]. Indeed, although only two of our ‘seed’ participants were under age 24, the weighted prevalence of youth (<24) in the sample was 33.6%. Our findings suggest that bisexual youth may have poorer mental health than bisexuals over age 24: the youth in our sample scored significantly higher on measures of depression and PTSD symptoms, and reported a significantly higher rate of past year suicidal ideation (29.7% vs. 15.2%). Although the difference was not statistically significant, the bisexual youth also reported more past-year suicide attempts (5.1% vs. 1.8%). As noted above, these rates of suicidal ideation and attempt in both youth and non-youth are higher than rates reported for the general population. Other studies of sexual minority youth have reported even higher rates of suicide attempts. For example, in one study of 11^th^ grade students, 21.5% of sexual minority youth and 4.2% of heterosexual youth reported a past year suicide attempt [55].

Although many studies have compared mental health outcomes between sexual minority and heterosexual youth, we could identify no other studies that specifically compared outcomes between youth and non-youth within a sexual minority sample. Sexual minority people often first recognize and/or disclosure their sexual orientation during adolescence and young adulthood, a time when financial and other independence is not yet achieved [56]–[57]. As discussed above, research consistently shows high rates of suicidality among sexual minority youth, and evidence suggests that such rates are impacted by supportive families and schools [58]. Bisexual youth may face particular challenges in disclosing their orientation and in finding acceptance and support because bisexuality tends to be more poorly understood, and is less likely to be treated as legitimate than lesbian or gay identity [59]. Indeed, a recent study of US population-based data from the National Longitudinal Study of Adolescent Health found that bisexuals were the only sexual identity group for which the percentage reporting suicide attempts did not decline significantly as participants moved into their late 20 s and early 30 s [60]. Additional research investigating potential relationships between bisexual identity acceptance, disclosure, and suicidality among both youth and adults would be warranted.

The findings of this study have important implications for researchers and practitioners working in the fields of public health and mental health, as well as those who work with youth in these and other settings. With respect to research, our findings of higher rates of poor mental health and greater substance use outcomes among bisexual people relative to rates for the general population underscore the need to study bisexuals as a discrete group (i.e., not collapsed together with other sexual minority people). Further, our finding of significant differences in outcomes between youth and non-youth suggests that age should be a variable of interest in future research on this topic. Finally, this is the first study we are aware of to use RDS to study bisexual people specifically. Our success in recruiting a large and diverse sample of bisexuals using this method suggests its potential for future studies in the field.

Our finding that outcomes—including depression, anxiety, and illicit drug use—are common among bisexuals indicates that mental health and substance use treatment services should be prepared to accommodate the specific needs of bisexual people. The little available research on mental health service experiences of bisexual people suggests that many providers lack understanding of bisexuality and how to address it in the context of mental health care [61]; we were not able to identify any studies focusing solely on the experiences of bisexual people who access treatment for substance use or addiction. As such, providers and administrators working in mental health/substance use service delivery may need to assess their capacity to meet the specific needs of this high-risk group.

Finally, our data suggest particular implications for those who work with youth in health, education, or other settings. The finding that youth are at elevated risk compared to non-youth for several of the outcomes examined, including past year suicidal ideation, suggests that bisexual youth may particularly benefit from resources that are supportive of their sexual identity. For example, many school settings have established “gay/straight alliances” to provide support for sexual minority students, and a relationship between protective school climate and reduced suicide risk has been established [62]. However, there is some evidence that these organizations may not always actively address the specific needs of bisexual youth [63]. Further, bisexual youth, more so than other sexual minority youth, may lack access to role models who share their sexual identity [64]–[65]. Programs designed to provide access to bisexual-specific role models, support, and information, should be evaluated to determine whether they could ameliorate the high rates of suicidality reported in this and other studies of bisexual youth.

In conclusion, the burden of mental health and substance use among bisexual individuals in Ontario is high relative to population-based studies of other sexual orientation groups. Bisexual youth in particular appear to be at risk for poor mental health. Additional research is needed to understand if and how minority stress explains this burden.
